# Tuberculous spondylodiscitis in abscess form

**DOI:** 10.1590/0037-8682-0581-2019

**Published:** 2020-03-16

**Authors:** Handan Alay, Elif Gözgeç

**Affiliations:** 1 Department of Infectious Diseases and Clinical Microbiology, Faculty of Medicine, Ataturk University, Erzurum, Turkey.; 2 Department of Radiology, Faculty of Medicine, Ataturk University, Erzurum, Turkey.

**FIGURE 1:**
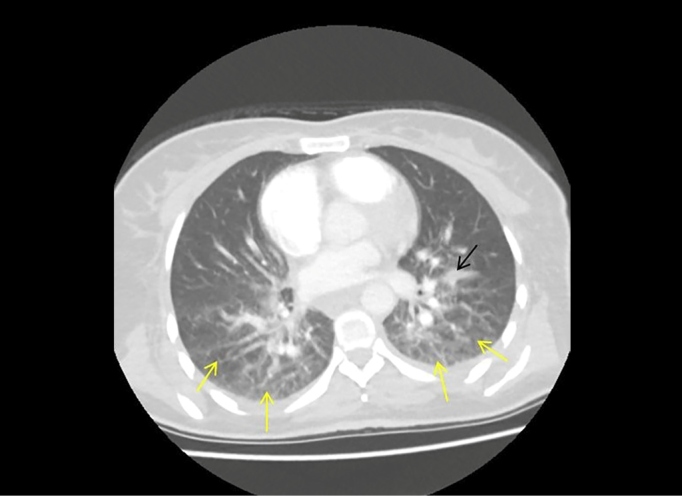
Diffuse complete-incomplete consolidation areas and increased reticulonodular thickness (yellow arrows) in the lower lobes of the bilateral lungs in axial thoracic computed tomography imaging. Fluid collection consistent with fissuritis visible in the left major fissure (black arrow)

**FIGURE 2:**
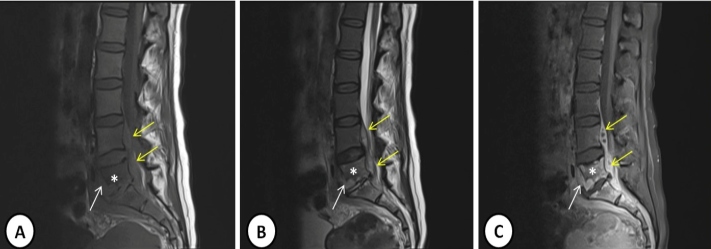
Lumbar magnetic resonance imaging consistent with an abscess at the L4-S2 vertebral level, appearing hypointense in T1 -weighted sections **(A)**, hyperintense in T2-weighted images **(B)**, and with intense contrast enhancement in post-contrast sections **(C)** (yellow arrows). Pathological signal changes compatible with spondylodiscitis exhibiting extension to the anterior epidural fatty planes are visible in the corners of the L5 vertebra (*) and S1 corpus and L5-S1 intervertebral disc intensity (white arrow).

**FIGURE 3:**
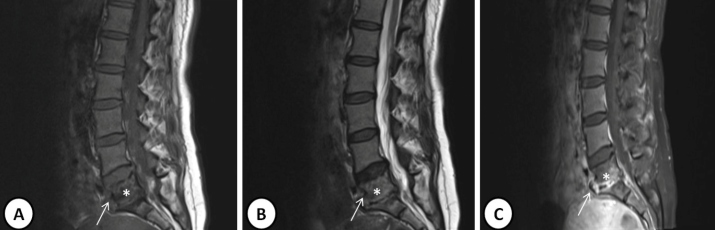
Post-treatment images showing narrowing in the L5-S1 discs in **(A)** T1-weighted, **(B)** T2-weighted **(B)**, and post-contrast images **(C)**. The white arrow showsdecreased pathological signals in the corners of the L5-S1 corpus exhibiting extension to the anterior epidural fat. (L5- *).

A 50-year-old woman was admitted to our clinic for low back pain, muscle spasm, and an inability to walk. She had attended different clinics due to low back pain for approximately one month without improvement in her symptoms. Physical examination revealed splenomegaly of approximately 1 cm and rales in the inferior lobes. The laboratory indicated a white cell count of 5.5 × 10^3^/µL, C-reactive protein concentration of 55.2 mg/dL, sedimentation rate of 79 mm/h, and tuberculin skin test measuring 18 mm. She tested negative for serum tube agglutination and autoantibodies positive for Quantiferon test, and negative for acid resistant-bacillus in her sputum. Pulmonary computed tomography showed ground-glass opacity and incomplete-complete consolidation in the bilateral lungs (Figure 1). A 1 × 9-cm abscess was observed at the L4-S2 vertebral level in lumbar magnetic resonance imaging (MRI), with contrast involvement in post-contrast sections (Figure 2). Specimens were not taken from the abscess material. The patient was started on four-drug anti-tuberculous therapy (isoniazid 300 mg/day, rifampicin 600 mg/day, ethambutol 2 g/day, pyrazinamide 2 g/day). Lesion regression was observed in control MRI performed at nine months (Figure 3). 

Spinal tuberculosis accounts for less than 1% of all tuberculosis cases^1^. Owing to MRI’s high sensitivity and specificity, it is a powerful diagnostic tool for the early diagnosis of tuberculous spondylodiscitis^2^. In cases where tuberculous agents cannot be grown in culture, appropriate radiological imaging methods should be applied. Thus,treatment can be initiated earlier and morbidity rates can be reduced.
